# Assessment and validation of glottic motion using cone-beam CT and real-time cine MRI

**DOI:** 10.1007/s00066-024-02204-y

**Published:** 2024-03-15

**Authors:** Seok-Joo Chun, Jaeman Son, Seonghee Kang, Chang Heon Choi, Jung-in Kim, Young-Il Kim, Joo Ho Lee, Jin Ho Kim, Hong-Gyun Wu

**Affiliations:** 1 Department of Radiation Oncology, Seoul National University Hospital, 101 Daehak-ro, 03080 Jongno-gu, Seoul, Korea (Republic of); 2 Biomedical Research Institute, Seoul National University Hospital, Seoul, Korea (Republic of); 3 Institute of Radiation Medicine, Seoul National University Medical Research Center, Seoul, Korea (Republic of); 4 Department of Radiation Oncology, Seoul National University College of Medicine, Seoul, Korea (Republic of); 5 Department of Radiation Oncology, Sejong Chungnam National University Hospital, Sejong, Korea (Republic of); 6 Cancer Research Institute, Seoul National University College of Medicine, Seoul, Korea (Republic of)

**Keywords:** Glottic cancer, Vocal cord, PTV margin, Radiotherapy, Cone-beam CT, ViewRay

## Abstract

**Purpose:**

This study aimed to assess the margin for the planning target volume (PTV) using the Van Herk formula. We then validated the proposed margin by real-time magnetic resonance imaging (MRI).

**Methods:**

An analysis of cone-beam computed tomography (CBCT) data from early glottic cancer patients was performed to evaluate organ motion. Deformed clinical target volumes (CTV) after rigid registration were acquired using the Velocity program (Varian Medical Systems, Palo Alto, CA, USA). Systematic (Σ) and random errors (σ) were evaluated. The margin for the PTV was defined as 2.5 Σ + 0.7 σ according to the Van Herk formula. To validate this margin, we accrued healthy volunteers. Sagittal real-time cine MRI was conducted using the ViewRay system (ViewRay Inc., Oakwood Village, OH, USA). Within the obtained sagittal images, the vocal cord was delineated. The movement of the vocal cord was summed up and considered as the internal target volume (ITV). We then assessed the degree of overlap between the ITV and the PTV (vocal cord plus margins) by calculating the volume overlap ratio, represented as (ITV∩PTV)/ITV.

**Results:**

CBCTs of 17 early glottic patients were analyzed. Σ and σ were 0.55 and 0.57 for left–right (LR), 0.70 and 0.60 for anterior–posterior (AP), and 1.84 and 1.04 for superior–inferior (SI), respectively. The calculated margin was 1.8 mm (LR), 2.2 mm (AP), and 5.3 mm (SI). Four healthy volunteers participated for validation. A margin of 3 mm (AP) and 5 mm (SI) was applied to the vocal cord as the PTV. The average volume overlap ratio between ITV and PTV was 0.92 (range 0.85–0.99) without swallowing and 0.77 (range 0.70–0.88) with swallowing.

**Conclusion:**

By evaluating organ motion by using CBCT, the margin was 1.8 (LR), 2.2 (AP), and 5.3 mm (SI). The margin acquired using CBCT fitted well in real-time cine MRI. Given that swallowing during radiotherapy can result in a substantial displacement, it is crucial to consider strategies aimed at minimizing swallowing and related motion.

## Introduction

Laryngeal cancer is a prevalent form of head and neck cancer, with approximately 1200 patients diagnosed and 300 patients dying from the cancer annually in Korea [1]. The majority of patients are diagnosed with early (T1-T2N0) cancer and disease originating from the glottic region. The standard treatment options for early glottic cancer include surgery or radiotherapy (RT), both of which have shown excellent outcomes in achieving local control rates of 80–90% for the disease [2, 3]. However, considering the goal of treatment for early glottic cancer, which involves maintaining both excellent local control and voice preservation, RT is often preferred [2, 4, 5].

Traditionally, RT was given in 60–70 Gy to whole laryngeal region with 2 Gy per fraction. However, technologic improvements such as intensity-modulated radiotherapy (IMRT) and stereotactic body radiotherapy (SBRT) have enabled hypofractionation trials aimed at reducing fraction size [4, 6–10]. As fraction size decreases, precise targeting of the tumor becomes increasingly crucial. To avoid mistargeting of tumors, margins from the clinical target volume (CTV) to the planning target volume (PTV) are given to compensate for interfractional organ movement, intrafractional organ movement, and setup errors. Immobilization using head support and a thermoplastic mask is routinely used to decrease possible setup errors. Additionally, image guidance using cone-beam computed tomography (CBCT) is used to overcome possible setup errors and organ movements. However, these techniques have limitations in reducing intra- and interfractional motion, as vocal cord motion is mainly due to breathing and swallowing. Especially swallowing during treatment can lead to significant mistargeting, as the movement of the vocal cords can be approximately 2 cm [11–17]. Furthermore, the vocal cords also experience interfractional deformation caused by breathing. Therefore, precise evaluation of vocal cord motion should be performed for the PTV margin. To address these concerns, our study aimed to evaluate these movements using real-time cine magnetic resonance imaging (MRI) and deformable CBCT.

## Methods

### Evaluating motion of vocal cords

For evaluation of intra- and interfractional motion, patients who were diagnosed with early glottic cancer (cT1-3N0) from 2020 to 2023 were recruited. All received the definitive aim of hypofractionated RT. In our institution, head rest and thermoplastic aquaplast were made individually to reduce setup errors. For the RT treatment, we generally employed simultaneous integrated boost (SIB) technique. The first CTV (CTV1) encompassed the gross tumor volume, while the second CTV (CTV2) encompassed the remaining larynx, excluding the posterior commissure unless it was in proximity to the gross tumor. Then, given a 3-mm margin, doses of 59.5 Gy and 47.6 Gy over a course of 17 fractions were prescribed to PTV1 and PTV2, respectively. Biweekly or daily CBCT was performed during the treatment. Therefore, all patients had five or more CBCTs. Patients were excluded if they had received definitive surgery such as total laryngectomy, were suspicious for lymph node metastasis, or had a previous history of head and neck RT.

**Fig. 1:**
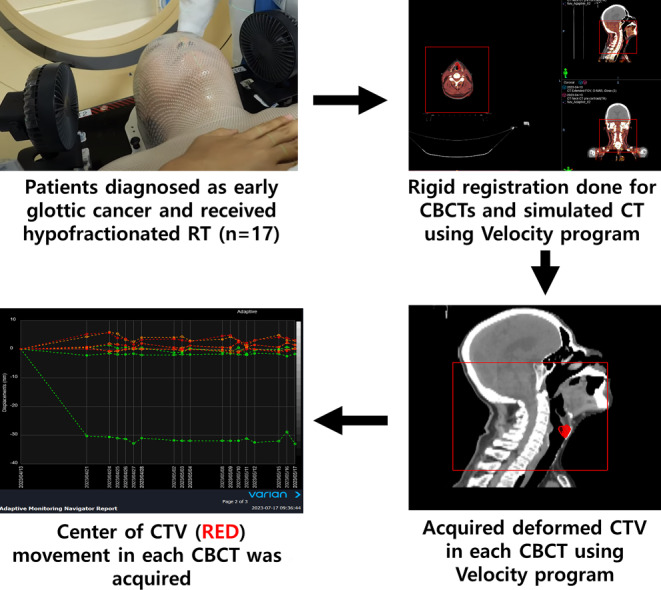
Evaluation of the margin for vocal cord using cone-beam computed tomography. *RT* radiotherapy, *CBCT* cone-beam computed tomography, *CT* computed tomography, *CTV* clinical target volume

We defined vocal cord motion as deformation of the vocal cord between CBCTs. We contoured the CTV as described above during the planning CT. Subsequently, to evaluate organ motion, we generated a deformed structure of the CTV for each CBCT scan using the Velocity program (Varian Medical Systems, Palo Alto, CA, USA). After deformation, we acquired all the movement changes at the center of the CTV for each CBCT, assuming it was organ motion. Figure 1 describes how the overall method was performed using the CBCT.

Subsequently, the systematic error and random error of movement were acquired from the changes at the center of the CTV. The systematic error was defined as the average movement in left–right (LR), craniocaudal (CC), and anterior–posterior (AP) directions. The random error was defined as standard deviation of movements. The population of systematic error (Σ) was evaluated from the standard deviation of all systematic errors and the population of random error (σ) from the root mean square of all random errors. Margins of intra- and interfractional were independently acquired using the Van Herk formula [18]: 2.5 Σ + 0.7 σ.

### Validation using real-time cine MRI and 4D planning CT

**Fig. 2:**
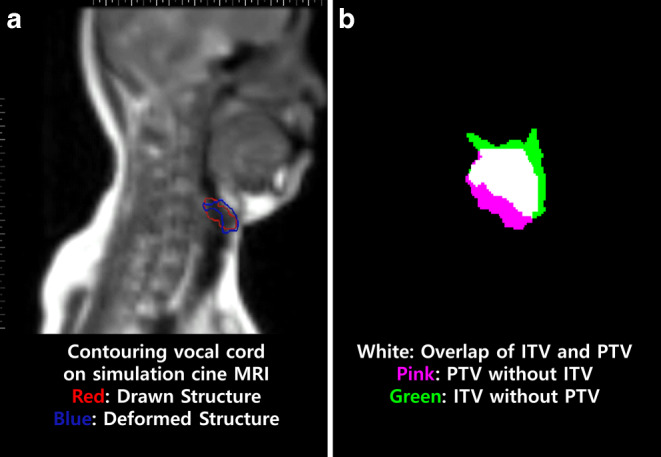
**a** Sagittal view from real-time cine MRI featuring contoured and deformed structures. The *red* structure represents the vocal cord outlined during simulation MRI, while the *blue* structure represents the real-time vocal cord structure deformed from the initial vocal cord, facilitated by the ViewRay system system (ViewRay Inc., Oakwood Village, OH, USA). **b** Volumetric representation of the ITV and PTV upon export to a DICOM file. The cumulative structure of the real-time vocal cord (*blue* structure from **a**) defines the ITV, while the PTV is formed by adding the margin to the simulation vocal cord (*red* structure from **a**). The ITV encompasses the total volume of white and green volumes and suggests a close similarity between ITV an PTV. *MRI* magnetic resonance imaging, *ITV* internal target volume, *PTV* planning target volume, *DICOM* Digital Imaging and Communication in Medicine

For validation, we prospectively recruited healthy volunteers for our study. All participants were given a full explanation of the study and informed consent was acquired. All participants exhibited no evidence of disease and were devoid of any laryngeal symptoms. The ViewRay treatment system (ViewRay Inc., Oakwood Village, OH, USA) is a radiation treatment machine that offers real-time cine imaging with its 0.35T MRI. During treatment simulation, we can assign treatment targets, which are deformed in real-time sagittal cine MRI. We defined the target as both left and right vocal cords (Fig. 2a). We acquired real-time imaging for 10–20 s in each target with four frames per second. After acquisition of real-time cine MRI, we imported it into a Digital Imaging and Communication in Medicine (DICOM) file. Subsequently, the margins for the vocal cord were applied, essentially considered as the PTV. Since we obtained exclusively sagittal MRI data, margins were assigned only for AP and SI directions. Moreover, considering the entirety of deformation-related movements observed during real-time MRI, we aggregated these movements to define the internal target volume (ITV). Figure 2b is a volume example of one participant in the study. To evaluate the extent of PTV coverage encompassing the ITV, we computed the overlap ratio by employing the following formula:

$$\text{Overlap Ratio}=\frac{PTV\cap ITV}{ITV}$$

The overlap ratio ranges between 0 and 1, where a value of 1 indicates complete coverage of the ITV by the PTV.

Furthermore, validation was performed using 4D planning CT. After the summer of 2023, we enrolled patients with early glottic cancer for 4D planning CT to evaluate potential interfractional motion during CT simulation. The gross tumor volume (GTV) was contoured in each phase from 0 to 90. The ITV was determined by summing up the total volume of the GTV in each phase. Subsequently, at the average phase, GTV was contoured and provided with the margin obtained using the Van Herk formula from CBCT. GTV with the margin was designated as the PTV, and the overlap ratio between ITV and PTV was evaluated using the aforementioned method with 4D planning CT.

### Ethics and statistics

Our study received approval from the institutional review board of our institution (IRB no.: 2205-089-1324). Informed consent was waived for patients with early glottic cancer, while it was obtained from healthy volunteers participating in the study. All analyses were conducted using R project version 4.2.3 (R Foundation for Statistical Computing, Vienna, Austria. URL https://www.R-project.org/). Student t‑test was used for evaluation of two continuous variables, while the Pearson correlation coefficient was used for evaluating a possible relationship between two variables. A *p*-value less than 0.05 was considered statistically significant.

## Results

A total of 17 patients with early glottic cancer were analyzed using CBCT. A total of 173 CBCTs were analyzed, varying from 5 to 17 for each patient. Σ and σ were 0.55 and 0.57 for LR, 0.70 and 0.60 for AP, and 1.84 and 1.04 for SI. Proposed margins using the Van Herk formula were 1.8 mm (LR), 2.2 mm (AP), and 5.3 mm (SI). Table 1 summarizes the results of motion errors and the margin measured in CBCT.

**Table 1:** Systematic errors, random errors, and calculated margin for left–right, anterior–posterior, and superior–inferior directions using cone-beam computed tomography

Error type	LR	AP	SI
Average error (µ, mm)	0.23	0.03	0.24
Systematic error (Σ, mm)	0.55	0.70	1.84
Random error (σ, mm)	0.57	0.60	1.04
Calculated margin (mm)	1.8	2.2	5.3

*LR* left-right, *AP* anterior-posterior, *SI* superior-inferior

**Fig. 3:**
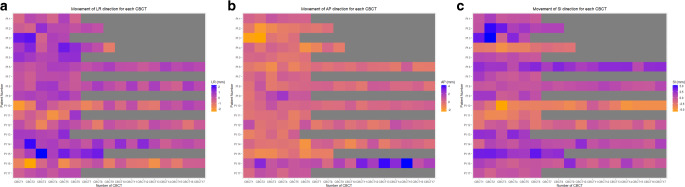
Heatmap of movement in **a** left–right, **b** anterior–posterior, and **c** superior–inferior direction in each cone-beam CT of 17 early glottic cancer patients. The Y‑axis represents individual patients, and the X‑axis corresponds to each cone-beam CE scan. Thus, each voxel represents the actual movement observed in each patient during their CBCT sessions. The *grey* voxels indicate cases where CBCT scans were not performed for specific patients. *LR* left–right (+ left, − right), *AP* anterior–posterior (+ anterior. − posterior), *SI* superior–inferior (+ superior, − inferior), *CBCT* cone-beam computed tomography

Out of the 17 patients, 6 individuals demonstrated movement surpassing the acquired margin at least once. In the SI direction, only two patients exhibited movement exceeding 5.3 mm, occurring only once per patient. Regarding the AP direction, 3 patients encountered movements exceeding 2.2 mm during eight sessions. Similarly, for the LR direction, 6 patients recorded displacements surpassing 1.8 mm in seven sessions. We inferred that calculating the margin for the SI direction would suffice, while greater margin consideration might be necessary for the AP and LR directions. As a result, we concluded that margins of 3 mm for AP and LR directions and 5 mm for the SI direction would be appropriate. Of a total of 173 sessions, three sessions of 3 patients exceeded 5 mm for the SI direction, while five sessions of 1 patient exceeded 3 mm for the AP direction, with no cases exceeding 3 mm for LR sessions. Figure 3 visually illustrates the displacements between initial CT and CBCT in three directions. Of note, 7 individuals exhibited an average movement exceeding 2 mm in the SI direction. In contrast, the average movements in LR and AP directions were relatively modest, with only 1 patient surpassing 1 mm, yet remaining below the 2 mm threshold.

**Fig. 4:**
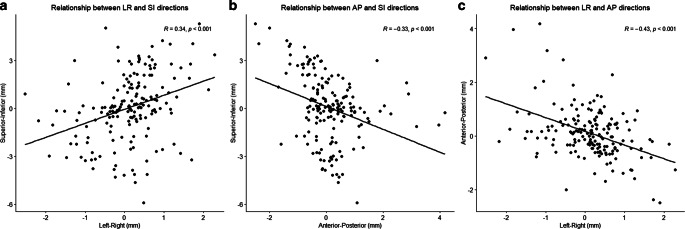
Correlation of **a** left–right and **b** superior–inferior and **c** left–right and anterior–posterior directions. Each point represents the movements evaluated by each cone-beam CT. *LR* left–right (+ left, − right), *SI* superior–inferior (+ superior, − inferior), *AP* anterior–posterior (+ anterior, − posterior)

Out of a total of 173 CBCT sessions, a clearly correlated tendency was observed in the left and superior positions (R = 0.34, *p* < 0.001). Conversely, an inverse correlation was evident in the anterior and superior directions (R = −0.33, *p* < 0.001), as well as the left and anterior directions (R = −0.43, *p* < 0.001). These movement tendencies are visually represented in Fig. 4, where each movement assessed by CBCT is depicted as a point on the graph.

To validate the proposed margin, a total of four healthy participants without a history of cancer were prospectively recruited. This resulted in the analysis of a combined total of eight vocal cords (including both left and right sides). For each vocal cord, PTV margins of 3 mm and 5 mm were applied in the AP and SI directions, respectively. The average overlap ratio, which was previously defined as the overlap volume of PTV and ITV divided by ITV, was 0.87 (range 0.70–0.99). Notably, out of the eight simulation MRI sessions, swallowing events were noted in three sessions (37.5%). When swallowing was observed, the overlap ratio was notably lower, at 0.77 (range 0.70–0.88). In contrast, a higher overlap ratio of 0.92 (range 0.85–0.99) was observed during sessions without swallowing events (*p* = 0.08). For the validation using 4D planning CT, analysis was conducted on four patients with early glottic cancer. Of note, a high overlap ratio was observed, with an average of 0.93 (range 0.92–0.95). Table 2 demonstrates all overlap ratios of eight participants.

**Table 2:** The overlap ratio of eight vocal cords for four participants using real-time cine magnetic resonance imaging

Participants No	Technique	Left VC	Right VC
1	ViewRay	0.88^a^	0.97
2	ViewRay	0.99	0.93
3	ViewRay	0.86	0.73^a^
4	ViewRay	0.85	0.70^a^
5	4D planning CT	0.93	
6	4D planning CT	0.94	
7	4D planning CT	0.92	
8	4D planning CT	0.95	

*No*. number, *VC* vocal cord, *CT* computed tomography

^a^Swallowing observed during simulation MRI

## Discussion

In this study, we assessed the calculated margin using the Van Herk formula in the context of CBCT scans of patients with early glottic cancer. The determined margins were 1.8 mm for the LR direction, 2.2 mm for the AP direction, and 5.3 mm for the SI direction. Moreover, considering instances where movements exceeded these calculated margins, we proposed an adjusted margin strategy of 3 mm for the AP and LR directions and 5 mm for the SI direction. To validate this approach, we employed ViewRay real-time cine MRI, revealing an overlap ratio of 0.87. Importantly, this ratio was notably higher in sessions without swallowing events (averaging 0.92) compared to sessions with swallowing events (averaging 0.77).

Several reports have been published regarding hypofractionated treatment of early glottic cancer [4, 7]. Two prospective studies have been reported, with conflicting results [6, 10]. Sher et al. observed that among 29 patients, dose-limiting toxicities were encountered in two active smokers, while the rest tolerated up to 42.5 Gy for 5 fractions [6]. On the contrary, Kang et al. reported early closure of their prospective dose-escalation trial due to two cases of grade 3 laryngeal inflammation with a SIB dose of 55 Gy and 40.7 Gy over 11 fractions [10]. Discrepancies between these results may be attributed to differences in manipulated target volumes. Sher et al. defined the CTV as the ITV plus 2 mm, while Kang et al. applied an SIB approach, encompassing the total larynx in the RT field. Larger treatments are related to higher doses to normal tissues, which can consequently lead to laryngeal toxicities [8]. Additionally, Sher et al. employed fiducial markers for respiratory tracking with four-dimensional CT to account for movements of vocal cord. These variations potentially contribute to the divergent findings witnessed in these contemporaneous clinical trials. In fact, we are currently trying to conduct a prospective multi-institutional trial for SBRT of early glottic cancer. A critical step in our preparatory phase involved the execution of this study, aimed at assessing the potential margins for the forthcoming trial. Notably, our investigation unveiled significant movement along the SI axis. This finding gains particular relevance given our institution’s adherence to a 3-mm PTV margin in all spatial directions.

Similar works using CBCT to evaluate the motion of vocal cords have been reported previously [19, 20]. Kwa et al. reported that the estimated margin for intrafractional motion using CBCT in 42 patients was 1.6 mm for LR, 4.3 mm for SI, and 2.2 mm for AP [20]. Additionally, the applied margin of 3 mm for AP and LR and 5 mm for SI was adequate, as all patients had at least 94% of the prescribed dose. Perillo et al. evaluated 23 patients with early glottic cancer who received 36 Gy in 3 fractions. Their study demonstrated similar results, i.e., 2.4 mm for LR, 5.1 mm for SI, and 2.2 mm for AP. We also report that the calculated margin was 1.78 mm for the LR, 2.16 for the AP, and 5.33 mm for SI direction, which were comparable to previous studies. Additionally, our study supports the use of margin of 3 mm for the AP and LR directions and 5 mm for the SI direction, which were commonly used margins in previous studies [6, 19, 20].

A notable limitation in using CBCT for evaluating vocal cord motion lies in its lack of real-time imaging capability, preventing an assessment of motion induced by swallowing. While multiple CBCT sessions can help capture potential intrafractional vocal cord motion, the dynamic influence of swallowing remains unaccounted for. On the contrary, cine MRI offers a noninvasive and ionizing radiation-free approach, comparable to gold standard methods like fluoroscopy or endoscopy for swallowing evaluation [15, 17]. Notably, Bradley et al. highlighted larynx motion of up to 3 mm in the SI direction even during rest, a factor that can be overlooked in CBCT analyses [15]. Addressing this limitation, we bolstered our study by corroborating the CBCT-derived margin using findings from participants undergoing real-time cine MRI. Through the combined application of CBCT and real-time cine MRI, our evaluation revealed the acquired margin to be satisfactory. However, as higher discrepancies between ITV and PTV were observed when swallowing, strategies aimed at reducing the impact of swallowing on treatment accuracy should be explored, to ensure the precision and efficacy of RT.

The larynx is known as a mobile organ, motion which is caused by breathing and swallowing. Although the thermoplastic mask is known to reduce possible setup errors and general movement, motion of the glottis cannot be prevented, especially in the SI direction. It is known that swallowing can cause severe vocal cord displacement of more than 2 cm [12, 16, 21]. Some efforts to reduce the effect of swallowing during RT have been reported [6, 11, 12, 19]. One potential approach involves providing patients with instructions to refrain from swallowing during the course of RT. Notably, such instructions have been shown to decrease the frequency of swallowing events compared to situations where no specific guidance was provided [12]. This parallels the findings of a study conducted by Perillo, which similarly incorporated instructions against swallowing during RT, and reported a 5-mm margin requirement for the SI direction [19]. An alternative strategy involves use of a gating system to mitigate potential mistargeting of the vocal cord induced by swallowing [6, 11]. Sher et al. initially employed an internal fiducial-based respiratory tracking system employing three fiducials. Due to challenges in tracking all fiducials consistently, they introduced additional skin fiducials to enhance accuracy [6]. Additionally, the same group proposed a surface-based gating technique, wherein real-time calculations of surface discrepancies are made using cameras [11]. Notably, our study aligns with their findings, revealing significant mistargeting of the vocal cord attributed to swallowing even after applying a 5-mm SI margin. In light of these results, it becomes imperative to consider measures aimed at mitigating the influence of swallowing in future prospective trials.

One of the limitations notably stems from the imaging and deformation capabilities of the ViewRay system. Due to its use of a 0.35‑T MRI for cine purposes, the attained image quality is inherently restricted. The process of deformation within the real-time MRI is also marred by errors attributed to the compromised image quality, potentially contributing to the observed reduction in the overlap ratio. Additionally, the intrinsic constraints of the real-time cine MRI, offering only a single sagittal image per simulation, restrict assessment of the LR direction. An additional limitation is the lack of representation of early glottic cancer patients due to the small sample size. While each methodology used in this study presents its unique limitations, the acquisition and validation of the margin for the vocal cord was successfully achieved through incorporation of two distinct methodologies.

In summary, the determined PTV margins for early glottic cancer were 1.8 mm for the LR, 2.2 mm for the AP, and 5.3 mm for the SI direction. Upon applying a 3-mm AP and 5‑mm SI margin, the observed overlap ratio proved satisfactory in sessions without swallowing events (average of 0.92), in contrast to sessions with swallowing events (average of 0.77). Given the impact of swallowing-induced movement, strategies to mitigate the influence of swallowing should be investigated in future studies.
